# The Effects of Trimetazidine and Sildenafil on Bilateral Cavernosal Nerve Injury Induced Oxidative Damage and Cavernosal Fibrosis in Rats

**DOI:** 10.1155/2014/970363

**Published:** 2014-03-18

**Authors:** Dogan Atilgan, Bekir S. Parlaktas, Nihat Uluocak, Fikret Erdemir, Fatma Markoc, Oguzhan Saylan, Unal Erkorkmaz

**Affiliations:** ^1^Department of Urology, Medical Faculty, Gaziosmanpasa University, 60100 Tokat, Turkey; ^2^Department of Pathology, Medical Faculty, Gaziosmanpasa University, 60100 Tokat, Turkey; ^3^Department of Biochemistry, Medical Faculty, Gaziosmanpasa University, 60100 Tokat, Turkey; ^4^Department of Biostatistics, Medical Faculty, Gaziosmanpasa University, 60100 Tokat, Turkey

## Abstract

*Aim*. The aim of this study was to compare the effects of sildenafil and trimetazidine on bilateral cavernosal nerve injury-induced oxidative damage and fibrotic changes in cavernosal tissue in rat model. *Material and Methods*. A total of 32 male Sprague-Dawley rats were randomly divided into 4 groups; each group consist 8 rats (control, BCI, BCI + TMZ, and BCI + sildenafil groups). Tissue superoxide dismutase (SOD), malondialdehyde (MDA), and protein carbonyl (PC) levels were determined biochemically and distribution of cavernosal fibrosis density among groups was performed histopathologically. *Results*. Tissue SOD levels in BCI group were significantly lower than the control group (*P* < 0.05). Tissue MDA and PC levels in BCI group were significantly higher than the control group (*P* < 0.05). TMZ and sildenafil administration significantly increased tissue SOD levels (*P* < 0.05) and reduced tissue MDA and PC levels (*P* < 0.05). Histologically, the degree of cavernosal fibrosis and collagen density was higher in BCI group in comparison to control, TMZ-treated, and sildenafil-treated groups. *Conclusion*. BCI caused oxidative damage and increased cavernosal fibrosis in rat penis. TMZ and sildenafil treatment decreased oxidative damage and reduced the degree of fibrosis in penile tissue due to BCI.

## 1. Introduction

The number of radical prostatectomy operations has increased worldwide as a result of the advances in the early diagnosis of prostatic carcinoma in recent years. Despite the advances in laparoscopic, robotic, and open radical prostatectomy techniques, postprostatectomy erectile dysfunction (PPED) already appears as one of the most important complications affecting patient's quality of life after radical prostatectomy. The ratio of PPED after bilateral nerve-sparing radical prostatectomy has been reported between 14% and 61% in the literature [1–3]. According to the related reports, the cavernosal nerve damage appears to be the most important factor in the development of ED after radical prostatectomy. A number of therapeutic interventions have been proposed to prevent PPED. One of these methods is long-term use of PDE5 inhibitors. In previous studies, it has been shown that PDE5 inhibitors reduced the formation of cavernosal fibrosis effectively, which led to prevention of PPED [4–7].

Oxidative injury (OI) is a complex phenomenon that causes destruction in both local and remote tissues [8]. The OI develops when there is excessive production of reactive oxygen species (ROS) and/or free radicals, which exceeds the natural antioxidant defence mechanisms in the body. The oxidative stress can produce important destructive effects in tissues by causing alterations in the cell membranes, leading to irreversible cellular damage. Eventually, OI causes increase in tissue levels of malondialdehyde (MDA) and protein carbonyl (PC), which are the end products of lipid peroxidation and protein oxidation, respectively [9].

The protective effects of trimetazidine (1-(2, 3, 4-trimethoxybenzyl)piperazine dihydrochloride) (TMZ) in OI have been shown in many studies. It showed antioxidant effects by partial inhibition of fatty acid oxidation, reduced ionic imbalance which occurred during OI, and prevented membrane damage due to oxidative stress. Additionally, it also has been shown that TMZ reduced intracellular acidosis, protected ATP synthesis, and limited inflammatory process and formation of ROS [10–15]. Neuroprotective effects of TMZ on the peripheral nerve damage were also reported in the literature [16].

In this study, we aimed to compare the effects of TMZ and sildenafil on the OI parameters in the cavernosal tissues of rats with bilateral cavernosal nerve injury and to evaluate the fibrotic changes in these tissues.

## 2. Materials and Method

### 2.1. Experimental Design

After the approval of the study design by the local ethical committee, a total of 32 male Sprague-Dawley rats were randomly divided into 4 groups. Group 1 (*n* = 8) was the control group; only penectomy was performed in this group. In Group 2 (*n* = 8), bilateral cavernosal nerve injury was constituted and penectomies were performed (BCI group). In Group 3 (*n* = 8), trimetazidine (TMZ) was given orally (5 mg/kg/day) for one week before BCI execution and continued 21 days after the operation (BCI + TMZ group). In Group 4 (*n* = 8), sildenafil was given orally (5 mg/kg/day) for one week before BCI execution and continued 21 days after the operation (BCI + sildenafil group). The penile tissues of the rats were analysed immunohistochemically and tissue oxidative damage parameters such as superoxide dismutase (SOD), malondialdehyde (MDA), and protein carbonyl (PC) levels were determined. In histopathological examination, the intensity of cavernosal fibrosis was graded and distribution among groups was performed.

*The Creation of Bilateral Cavernosal Nerve Injury (BCI).* Under xylazine anaesthesia (10 mg/kg i.p.), the rats were fixed to the table in supine position and the area was exposed via suprapubic median incision. The cavernosal nerves were identified at the posterolateral region of the prostate bilaterally. In consistence with the previous studies, the nerves were clamped for about 2 seconds to create neural injury. This procedure was applied to the rats in Groups 2, 3, and 4. On the 28th day of the total experimental time and 21st day of BCI execution, penectomies of the rats were performed. Penile tissues were sent to laboratory for biochemical and histopathological examination in formaldehyde solution.

### 2.2. Biochemical Analyses

Homogenization procedure of the penile tissues was performed for 2 min at 13,000 rpm with the homogenizer (IKA Ultra-Turrax t 25 Basic, Germany) in five volumes of ice-cold Tris-HCl buffer (50 mM, pH 7.4) containing 0.50 mL/L Triton x-100. Subsequently, determinations were carried out on the prepared homogenate, supernatant, and extracted samples using commercial chemicals produced by Sigma (St. Louis, USA). In compliance with the description of the procedure in previous studies, the protein measurements were executed in the samples.

#### 2.2.1. Determination of Tissue Superoxide Dismutase (SOD) Activity

The method of Sun et al. was used in the determination of SOD activity [17]. The inhibition of nitroblue tetrazolium (NBT) reduction by xanthine-xanthine oxidase system as a superoxide generator was the basic principle of the method. After adding 1.0 mL of ethanol-chloroform mixture (5/3, v/v) to the same volume of sample and centrifugation, the activity of SOD was assessed in the ethanol phase of the supernatant. The amount causing 50% inhibition in the NBT reduction rate was accepted as one unit of SOD and the activity of SOD was presented as U/mg protein for penile tissue.

#### 2.2.2. Determination of Malondialdehyde (MDA) Level

The level of the thiobarbituric acid-reactive substance was determined by using the method which is based on reaction with thiobarbituric acid (TBA) at 90 to 100°C [18]. In this TBA test reaction, MDA and TBA enter into a reaction producing a pink pigment which is absorbed maximally at 532 nm. The reaction was performed at 90°C for a period of 15 min in a pH = 2-3 media. After mixing the sample with two volumes of cold 10% (w/v) trichloroacetic acid to precipitate the protein, this precipitated protein was pelleted by centrifugation. After the reaction of an aliquot of the supernatant with an equal volume of 0.67% (w7V) TBA for about 10 min in boiling water, it was cooled and the absorbance was evaluated at 532 nm. The penile tissue MDA levels were presented as nmol/g wet tissue and graded according to the standard graphics which were formed by measurements with the standard solution (1, 1, 3, 3-tetramethoxypropane).

#### 2.2.3. Determination of Tissue Protein Carbonyl (PC)

The tissue PC content was determined by using a spectrophotometer (Cintra 10 E, Austria), which measured the quantity of the reaction of carbonyl group with 2, 4-dinitrophenylhydrazine to form 2, 4-dinitrophenylhydrazone [19].

### 2.3. Histopathological Examination

The penectomy materials were fixed in 10% formalin solution, subsequently washed, and stored in 70% alcohol at 4°C until paraffin-embedded tissue sectioning (5 *μ*m) process. The corpora cavernosal tissues were stained with Masson's trichrome. The collagen density in the connective tissue of cavernosal tissue was evaluated and graded on scales ranging from 1+, lowest density collagen, to 4+, highest density collagen ratios under light microscopy (×400).

### 2.4. Statistical Analysis

Biochemical and histopathological parameters among groups were compared with Kruskal Wallis analysis of variance. For multiple comparisons, Bonferroni adjusted Mann-Whitney  *U*  test was used. Biochemical and histopathological data were presented as the mean ± standard deviation. The *P* values of <0.05 were considered statistically significant. Analyses were performed using commercial software (IBM SPSS Statistics 19, SPSS Inc., an IBM Co., Somers, NY).

## 3. Results

### 3.1. Biochemical Analyses

The tissue SOD, MDA, and PC analysis results of all the groups were presented in Table 1. While tissue SOD levels were significantly lower in BCI group than control group (*P* < 0.001), tissue MDA and PC levels in BCI group were significantly higher in comparison with control group (*P* < 0.001). According to these results, it might be thought that BCI created oxidative stress in the rat cavernosal tissue. On the other hand, in BCI + TMZ and BCI + sildenafil groups, tissue SOD levels were significantly higher than BCI group (*P* < 0.001). Tissue MDA and PC levels were significantly lower in BCI + TMZ and BCI + sildenafil groups compared to BCI group (*P* < 0.001). In terms of tissue SOD, MDA, and PC levels, there were no significant differences between group 1, group 3, and group 4 (*P* > 0.05). These findings indicated that TMZ and sildenafil treatment reduced BCI-induced oxidative damage in rat cavernosal tissue.

### 3.2. Histopathological Evaluation

**Figure 1:**
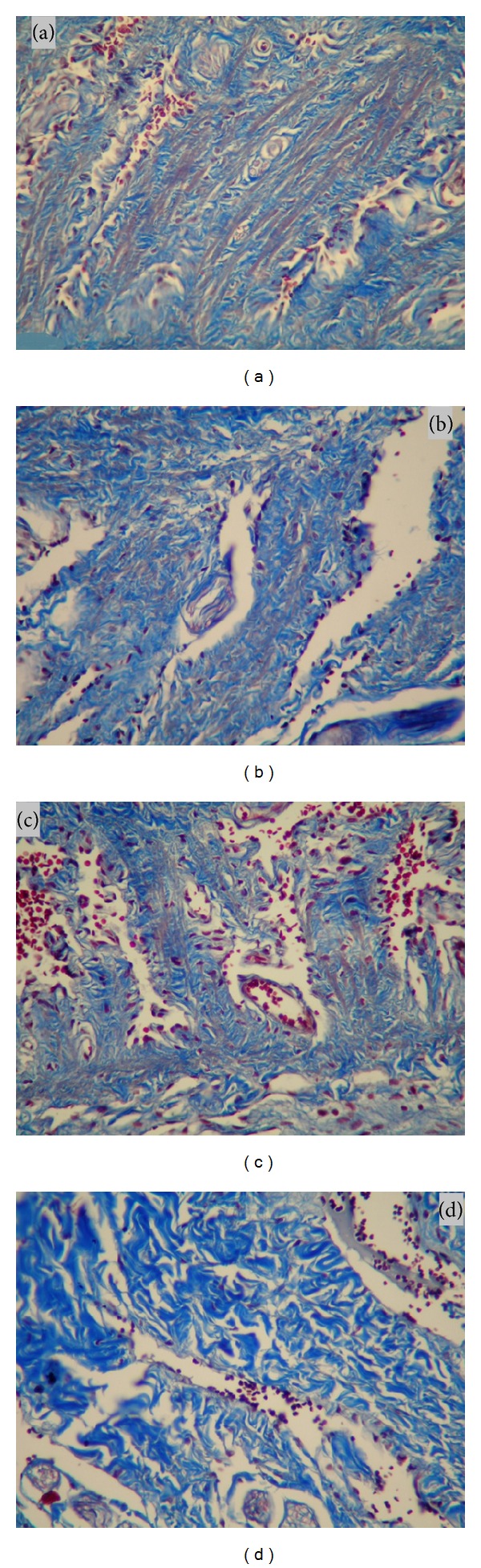
Histological view of collagen density in the cavernosal tissues stained with Masson's trichrome (×400).

Histopathological distribution of tissue collagen densities was presented in Table 2. The distribution of collagen densities in BCI group was more intense than the control, TMZ-treated, and sildenafil-treated groups. None of the rat penile tissues in control, TMZ, and sildenafil groups have high density of collagen, whereas high collagen density was detected in 4 rats of BCI group. Additionally, in control, TMZ-treated, and sildenafil-treated groups, collagen density distributions were observed in mild and moderate levels (Figure 1).

## 4. Discussion

Despite the different nerve-sparing techniques, erectile dysfunction after nerve-sparing radical prostatectomy has not been determined, yet. Many theories have been proposed about the formation of ED after nerve-sparing radical prostatectomy. The stretching of nerve, direct neural injury, pelvic fibrosis, and nerve entrapment due to surgery may play role in the etiopathogenesis of PPED. Due to cavernosal nerve damage, a pathophysiological process occurs in cavernosal tissue and, as a result of this process, smooth muscle/collagen ratio changes in favour of collagen. Eventually, fibrosis formation occurs in cavernosal tissue [20]. In previous experimental studies, it has been showed that bilateral cavernous nerve injury causes OI which led to varying degrees of apoptosis in smooth muscle cells and fibrosis in cavernosal tissue [21]. Klein et al. demonstrated apoptosis in the erectile tissues of the rat penis following denervation [22]. In a recent study, User et al. has also demonstrated formation of apoptotic smooth muscle cell population in the subtunical location in denervated rat penis [23]. This functional loss in the subtunical smooth muscle cells may contribute to a deficient venoocclusive mechanism, thus leading to ED.

The relationship between oxygen concentration and cavernosal fibrosis has also been shown in several studies. Moreland et al. showed that TGF-b1 increased collagen synthesis in human corpus cavernosal smooth muscle cells in culture which was induced by hypoxia [24]. In addition, Daley et al. reported that TGF-b1 induced collagen synthesis in human corpus cavernosum smooth muscle and prostaglandin E1 (PGE1) suppressed this process. They also noted that production of prostaglandin E1 (PGE1) was oxygen dependent [25]. In another study, Moreland observed that hypoxia induced TGF-b1 expression and inhibited PGE1 synthesis [26]. Fischer et al. noted that 3–5 nocturnal erection episodes per night occurred in men with normal erectile function and each lasting about 30–45 minutes [27]. The blood pO2 in the corpus cavernosum increases during these erectile events and this situation helps the synthesis of prostanoids and nitric oxide. In other words, the production of these substances is favoured by a higher oxygen tension. However, the loss of nocturnal erections due to nerve injury induced hypoxia in rat cavernosal tissue. Leungwattanakij at al. demonstrated an increase in TGF-b1, hypoxia inducible factor 1a (HIF-1a), and collagen III synthesis in rat cavernosal tissue after cavernous neurotomies [28]. Similarly, in our study, we determined that bilateral cavernosal nerve injury created a hypoxic environment in cavernosal tissue and this situation led to an oxidative damage in cavernosal tissue of rats.

The development of ED after nerve-sparing radical prostatectomy due to oxidative damage has been shown in numerous studies in the literature [29, 30]. According to these studies, oxidative stress due to surgical trauma caused accumulation of ROS in the tissues which caused detrimental effects on erectile function. The possible pathogenesis of OI on erectile dysfunction was hypothesized as the increased inactivation of NO by free radicals which resulted in impaired penile NO transmission and smooth muscle relaxation [31].

Recent studies have reported that long-term use of PDE5 inhibitors showed effectiveness among patients who had complaints of ED after radical prostatectomy [32, 33]. There are also some animal studies in the literature which showed the beneficial effects of PDE5 inhibitors on ED [34, 35]. The protective effects of sildenafil citrate on OI have been shown previously in many investigations related to I/R injury. This effect of sildenafil could be associated with its inhibiting effect on the production of lipid peroxidation via the activity and expression of nicotinamide adenine dinucleotide phosphate-oxidase [36–38]. In our study, we also determined that sildenafil treatment has significantly improved OI parameters in comparison with BCI group.

The protective effect of TMZ on OI also has previously been shown in many investigations in other tissues [39–41]. Additionally, in a recent study, it has been shown that TMZ administration had beneficial effects on sciatic nerve injury by inducing an increase in serum GSH level and decrease in serum NO and MDA levels and treatment with TMZ reversed the injury-induced deleterious effects on the number of myelinated axons in the sciatic nerve of rats [16]. In our study, we found that OI parameters in TMZ-treated group were significantly improved than BCI group and, to our knowledge, this is the first study in the literature that shows beneficial effects of TMZ against OI in cavernosal tissues.

In our study, we found that, in TMZ- and sildenafil-treated groups, SOD, MDA, and PC levels which were the indicators of OI were significantly improved compared with BCI group. Histopathologically, collagen density in TMZ- and sildenafil-treated groups was reduced in comparison to BCI group, as well.

Although this is an experimental preclinical study, the results of this study should be supported with more comprehensive and detailed further clinical studies. Nowadays, PDE5 inhibitors are being used in penile rehabilitation of PPED but, in the light of these future clinical studies, it may be speculated that the addition of TMZ to this treatment protocol will help in reducing the regeneration time of injured cavernosal nerves and PPED rates.

## 5. Conclusion

As a result of our study, BCI created oxidative stress in rat penile tissue. Consequently, cavernosal tissue collagen densities were increased due to oxidative injury induced by BCI and this may lead to development of fibrosis in the cavernosal tissues. But the use of TMZ and sildenafil decreased oxidative damage and reduced fibrotic process in rat penile tissues due to cavernosal nerve injury.

## Figures and Tables

**Table 1:** Tissue SOD, MDA, and PC levels of the groups.

	Control (*n* = 8)	BCI (*n* = 7)	BCI + TMZ (*n* = 7)	BCI + sildenafil (*n* = 7)	*P*
SOD (U/mg protein)	101.74 ± 9.34	11.12 ± 0.92^a^	83.20 ± 21.33	95.14 ± 13.59	**0.001**
MDA (nmol/g wet tissue)	0.54 ± 0.36	2.26 ± 0.42^a^	0.86 ± 0.37	0.36 ± 0.17	**<0.001**
PC (nmol/mL tissue)	611.77 ± 165.53	1256.94 ± 451.53^a^	706.03 ± 151.40	469.45 ± 113.44	**<0.001**

Data were presented as mean ± standard deviation.

^a^There was statistically significant difference from other groups (*P* < 0.05).

SOD: superoxide dismutase.

MDA: malondialdehyde.

PC: protein carbonyl.

BCI: bilateral cavernosal nerve injury.

TMZ: trimetazidine.

**Table 2:** Distribution of cavernosal collagen density in the groups.

Degree of collagen density				
	1+ (*n*)	2+ (*n*)	3+ (*n*)	4+ (*n*)
Group 1	5	3	0	0
Group 2	0	2	2	4
Group 3	2	4	2	0
Group 4	2	6	0	0

1+: lowest collagen density.

2+: mild collagen density.

3+: moderate collagen density.

4+: highest collagen density.
